# Homogeneity of cognitive and behavioural processes underlying the relationship between insomnia and body image disturbance

**DOI:** 10.1007/s10339-021-01039-0

**Published:** 2021-06-25

**Authors:** Umair Akram, Sarah F. Allen, Jodie C. Stevenson, Lambros Lazuras, Millicent Ackroyd, Jessica Chester, Jessica Longden, Chloe Peters, Kamila R. Irvine

**Affiliations:** 1 Department of Psychology, Sheffield Hallam University, Collegiate Crescent, South Yorkshire, Sheffield, S10 2BP UK; 2 School of Social Sciences, University of Teesside, Middlesbrough, UK; 3 School of Psychology, University of Lincoln, Lincoln, UK; 4 Department of Psychology, University of Derby, Derby, UK

**Keywords:** Sleep, Body image disturbance, Selective attention, Appearance fixing, Rationale acceptance

## Abstract

Specific cognitive behavioural mechanisms related to selective attention, situational avoidance and physical appearance are implicated in the development and maintenance of insomnia and negative reinforcement of body image disturbances. Therefore, we examined whether these processes potentially mediate the relationship between insomnia and body image perception. *N* = 728 participants completed self-reported measures of sleep-associated monitoring, insomnia symptoms, body image disturbance and coping with body image challenges. Symptoms of insomnia and sleep-associated monitoring behaviour were independently related to increased reports of body image disturbance, cognitive distortions of body image, appearance fixing (i.e. altering appearance by covering, camouflaging or correcting the perceived defect), avoidance (i.e. attempt to escape or avert stressful body image situations) and reduced levels of positive rationale acceptance (i.e. acceptance of the challenging event and positive self-care or rationale self-talk about one’s appearance). More crucially, sleep-related monitoring on awakening, cognitive distortion of body image and negative coping strategies related to body image (i.e. appearance fixing, avoidance, rationale acceptance) mediated the relationship between reports of body image disturbance and insomnia symptoms. The current findings expand upon previous research demonstrating consistent relationships between poor sleep and increased dissatisfaction with cutaneous features, by providing novel evidence that body image disturbances are associated with symptoms of insomnia. More crucially, we highlight the role of particular cognitive and behavioural mechanisms pertaining to sleep (i.e. selective attention for physical signs of poor sleep) and body image (i.e. avoidance and rationale acceptance) which may be targeted as part of cognitive behavioural treatments.

## Introduction

Symptoms of insomnia (including difficulty initiating and/or maintaining sleep, or early morning awakenings with an inability to return to sleep, and impaired daytime functioning) are highly prevalent, affecting 30% of the general population (American Psychiatric Association 2013; Espie et al. 2012; Morin et al. 2006). It has been evidenced that individuals experiencing insomnia frequently present with: (i) difficulties with emotion regulation and self-perception (Akram et al. 2016; Baglioni et al. 2010; Ypsilanti et al., 2018) and (ii) self-focused attention on awakening, as well as throughout the day, to assess and confirm the perceived poor nature of their sleep (Semler and Harvey 2005). According to cognitive models of insomnia (e.g. Harvey 2002), this pattern of thinking may perpetuate and maintain insomnia by potentiating cognitive arousal, distress and negative thoughts concerning sleep. This, in turn, further increases the likelihood of attentional allocation towards cues of sleep loss which may promote an insomnia-consistent interpretation of physical appearance.

Previous research demonstrates that this might be the case. For example, individuals with insomnia and poor sleepers report dissatisfaction with their cutaneous body image (a dimension of body image concerning one’s skin, hair and nails) and aspects of their facial appearance (Gupta et al. 2015; Oyetakin-White et al. 2015), suggesting that cutaneous body image dissatisfaction appears to be directly related to insomnia symptoms (Akram et al. 2017, 2020; Gupta et al. 2015). Additionally, poor sleepers present greater dissatisfaction with their facial appearance (e.g. complexion, skin age) when compared with normal sleepers (Oyetakin-White et al. 2015). A number of mechanisms may explain these relationships. Sleep promotes blood flow to the skin and changes in skin blood coloration occur with chronic sleep loss, leading the face to appear paler (Krauchi and Wirz-Justice 2001). This can be visually projected by alterations in dermatological characteristics (e.g. wrinkles/fine lines and bags around the eyes, dropped corners of the mouth and heavy eyes) pertaining to tiredness (Knoll et al. 2008; Sundelin et al. 2013). Indeed, photographs of sleep-deprived individuals have previously been rated as less attractive, less healthy, more tired and less sociable when compared to photographs of well-rested individuals (Axelsson et al. 2010; Sundelin et al. 2013).

Additionally, it is well established that individuals with insomnia also monitor bodily sensations on waking and during the day for physical signs of a poor night’s sleep, fatigue and tiredness (e.g. sore head, heavy eyes, poor facial complexation; Semler and Harvey 2004). During such behaviour, increased attention towards the physical self may contribute to body image dissatisfaction. Body dissatisfaction is one of the many dimensions of body image. The concept of body image is multi-dimensional, comprising attitudinal, affective, perceptual and behavioural dimensions (Cash and Deagle 1997; Gardner 2011). Body image disturbance (BID) refers to dysfunctional self-oriented attitudes (cognitive-evaluative dysfunction) and behaviours specifically related to disliked aspect of one’s own body (Cash et al. 2004, 2002). It (non-exhaustively) comprises body-related emotions and feelings, as well as thoughts and beliefs, such as body dissatisfaction, excessive self-evaluation of appearance, adverse emotions associated with body image, and appearance-focused comparison and/or making of undesirable psychical attributes of the self (Cash and Smolak 2011; Cash and Grasso 2005; Castle et al. 2002; Vossbeck-Elsebusch et al. 2015). Individuals presenting with BID often engage in specific cognitive and behavioural coping mechanisms which include: appearance fixing (i.e. altering appearance by covering, camouflaging or correcting the perceived defect), avoidance (i.e. attempt to escape or avert stressful body image situations) and reduced levels of positive rationale acceptance (i.e. acceptance of the challenging event and positive self-care or rationale self-talk about one’s appearance) (Cash and Smolak 2011). In turn, an increase in maladaptive coping strategies including excessive appearance fixing, avoidance of stressful body image situations (e.g. social situations) and diminished rationale acceptance may possibly further exacerbate negative self-appraisal.

It appears that cognitive behavioural mechanisms (e.g. selective attention, appearance fixing, situational avoidance) play crucial roles in both perpetuating insomnia and negative reinforcement of body image disturbances (Cash 2002; Harvey 2002). Therefore, these processes potentially mediate the relationship between insomnia and body image perception. However, whilst early steps have been taken to understand the role of poor sleep/insomnia in cutaneous body image, the potential relationships between poor sleep/insomnia and other aspects of body image (such a body image dissatisfaction, distress, and dysfunction) remain unclear. Therefore, this study examined the relationship between additional, more physical (body-related as opposed to cutaneous) aspects of body image and insomnia symptoms, whilst examining the mediating roles of: sleep-associated monitoring on awakening and throughout the day; cognitive distortion; and coping strategies for body image disturbance. More specifically, we examined whether: a) symptoms of insomnia would be related to increased reports of body image disturbance and b) engaging in sleep-associated monitoring, cognitive distortion of body image and negative coping strategies (i.e. appearance fixing, avoidance, rationale acceptance) would mediate the association between body image disturbance and insomnia symptoms.

## Methods

### Sample and procedure

The protocol was approved by the Sheffield Hallam University Research Ethics Committee. A cross-sectional online questionnaire-based study was implemented comprising questions designed to examine levels of sleep-associated monitoring, insomnia symptoms and body image disturbances. Students from three UK universities were recruited through institutional course participation schemes, social media and faculty emails. Members of the general population were recruited using social media platforms and online forums. Potential participants responded to an advertisement for a study examining the relationship between “Sleep and Self-Perception”. Overall, 877 participants began or accessed the questionnaire; after incomplete entries were discarded, 728 completed entries (mean age = 28.81 ± 12.59 years, range 19–75, 89% female; 66% student population; final response rate = 83%) were retained for analysis. This sample size was sufficient for a 95% confidence level, exceeding our target of 500 responses leaving an acceptable 4.5% margin of error (Niles 2006).

### Measures

#### Insomnia symptoms

Insomnia symptoms were assessed using Sleep Condition Indicator (SCI), a clinical screening tool developed to appraise insomnia symptoms against the DSM-5 criteria for Insomnia Disorder (Espie et al. 2014). The scale consists of eight items each scored between 0 and 4 designed to examine insomnia symptomology during the last month. Specifically, questions pertain to sleep onset latency, awakenings during the night, perceived sleep quality, impairment to daytime functioning and symptom persistence. Items are summed to create a total score between 0 and 32, with lower scores indicating greater insomnia symptom severity. Assessment of internal consistency in the present sample yielded an acceptable Cronbach’s alpha level of *α* = 0.87.

#### Sleep-associated monitoring

Monitoring (selective attention) was assessed using two subscales (daytime monitoring for body sensations: DM; monitoring for body sensations on awakening: WM) from the Sleep-Associated Monitoring Index (SAMI: Semler and Harvey 2004). Items are measured on a five-point scale on which participants indicate what is true for them over the past month (1 = not at all, 5 = all the time). Mean scores for subscales reflect total subscale scores divided by the number of items in the scale. Each subscale is comprised of five items. For the DM subscale, participants are asked how often they are aware of the following throughout the day: arms and/or legs feeling tired or heavy; muscle aches, cramps or pain; shoulders, neck or back feeling tense or sore; feelings of tension or discomfort in body; and stiffness in body, whereas for the WM subscale, participants are asked to report the extent to which they notice: feelings of tiredness or heaviness in the body; heaviness, soreness or itchiness in the eyes; arms or legs feeling tired or heavy; feeling fatigued; and feelings or sensations caused by sleep deprivation. Higher mean scores for each subscale represent increased monitoring for physical cues and sensations which may be attributed to poor sleep. Assessment of internal consistency in the present sample yielded a Cronbach’s alpha of *α* = 0.91 for DM and *α* = 0.89 for WM.

#### Body image

Three measures examined participants’ body image: the Assessment of Body Image Cognitive Distortions Form A (ABCD: Jakatdar et al. 2006); the Body Image Disturbance Questionnaire (BIDQ: Cash et al. 2004); and the Coping with Body Image Challenges Inventory (BICSI: Cash et al. 2005).

The Assessment of Body Image Cognitive Distortions (Jakatdar et al. 2006) is an 18-item self-report inventory that measures the tendency to engage in appearance focused cognitive distortions, i.e. how a person processes information about their physical appearance. Items explore hypothetical situations and thoughts related to appearance (e.g. Think about those aspects of your appearance that you’ve wished were different. Do you ever think that your future will be less satisfying because of how you look?). Participants rate each item using a five-point scale, ranging from 0 (not at all like me) to 4 (exactly like me). Total scores represent the mean of all items, with higher scores indicating the increased presence cognitive distortions in the context of appearance. Two parallel forms of the measure are available. Form A was used for the present study, and the internal consistency yielded in the present study was a Cronbach’s alpha of *α* = 0.96.

The BIDQ (Cash and Grasso 2005) is a seven-item measure of body image disturbance (conceived as body image dissatisfaction, distress and dysfunction). Items examine appearance-related concerns (e.g. Are you concerned about the appearance of some part(s) of your body which you consider especially unattractive?), preoccupation with such concerns (e.g. If you are at least somewhat concerned, do these concerns preoccupy you? That is, you think about them a lot and they’re hard to stop thinking about?), experience emotional distress (e.g. Has your physical “defect” often caused you a lot of distress, torment, or pain?) and impairments in social, occupational and other areas of functioning (e.g. Has your physical “defect” caused you impairment in social, occupational or other important areas of functioning?). Participants rate each item using a five-point scale, ranging from 1 to 5, with higher scores indicating the increased presence of body image disturbance symptoms. The mean across items represents the final score. In the present study, the internal consistency (Cronbach’s alpha) of the scale was *α* = 0.90.

Finally, the BICSI (Cash et al. 2005; Hrabosky et al. 2009) is a 29-item measure of how individuals cognitively and behaviourally cope with circumstances that threaten body image. On a four-point response format ranging from 0 (definitely not like me) to 3 (definitely like me), higher scores indicate greater likelihood of engaging in particular coping strategies. The BICSI consists of three subscales: Appearance fixing (BICSI-AF) includes attempts to change one’s appearance through concealing, camouflaging or correcting perceived imperfections (e.g. I fantasize about looking different). Avoidance (BICSI-AV) is the attempt to avoid or escape distressing body image situations or experiences (e.g. I withdraw and interact less with others). Positive rationale acceptance (BICSI-RA) entails strategies of acceptance of the event or associated thoughts or emotions, as well as managing them with positive self-care or rationale self-talk (e.g. I tell myself that there are more important things than what I look like). The internal consistency (Cronbach’s alpha) in the present sample was as follows: appearance fixing *α* = 0.87); avoidance *α* = 0.77; and positive rationale acceptance *α* = 0.86.

### Data analyses

SPSS (version 24, IBM Corp.) was used to perform formal statistical analyses, with significance considered at the p < 0.05 level. Correlational analyses (Pearson’s bivariate) examined the relationship between insomnia, sleep-associated monitoring and aspects of body image disturbance. This was followed by a hierarchical linear regression analysis (using the enter method) that empirically examined the multivariate association between symptoms of insomnia and body image disturbance after accounting for age, sex, sleep-associated monitoring and body image-related cognitive distortions and coping. Regression-based multiple mediation modelling was used with the SPSS PROCESS macro by Preacher (2007), to examine the indirect association between body image disturbance and insomnia symptoms, via sleep-associated monitoring and body image-related distortions and coping processes.

## Results

The mean scores for the final sample are presented in Table 1. Insomnia symptoms were significantly related to increased levels of sleep-associated monitoring on awakening, sleep-associated monitoring through the day, body image cognitive distortion, body image disturbance, appearance fixing, avoidance and reduced acceptance. All correlations are presented in Table 1.

**Table 1:** Descriptive statistics and correlation matrix for measures of sleep-associated monitoring, insomnia symptoms and body image disturbance variables

	Mean ± SD	1	2	*3*	4	5	6	7
1. Insomnia symptoms	16.54 ± 7.60							
2. Monitoring: awakening	2.96 ± 1.02	− 64*						
3. Monitoring: daytime	2.67 ± 1.05	− 60*	0.75*					
4. Body image cognitive distortion	2.02 ± 1.06	− 38*	0.36*	0.38*				
5. Body image disturbance	2.32 ± 0.94	− 42*	0.41*	0.39*	0.72*			
6. Coping: appearance fixing	1.62 ± 0.67	− 0.21*	0.25*	0.24*	0.69*	0.58*		
7. Coping: avoidance	1.30 ± 0.62	− 0.41*	0.40*	0.42*	0.62*	0.58*	0.46*	
8. Coping: rationale acceptance	1.31 ± 0.58	0.19*	− 0.13*	− 0.13*	− 0.23*	− 0.22*	− 0.02	− 0.04

*Sig at < 0.001

A bootstrapped (1000 resamples) hierarchical linear regression analysis was used to evaluate the direct and multivariate association between body image disturbance, insomnia symptoms, body image cognitive distortion, coping processes and sleep-related monitoring, after controlling for the effects of demographic variables (age and sex). The analysis was completed in two steps, with demographics and insomnia symptoms added in the first step and body image cognitive distortions, body image coping processes and sleep-related monitoring added in the second step of the analysis. The overall model was statistically significant (F = 117.95, *p* < 0.001) and predicted 59.3% (adjusted *R*^2^) of the variance in body image disturbance with a large multivariate effect size, *f*^2^ = 1.45, according to Cohen (1988). Tolerance levels were inspected for collinearity, and the lowest values were at acceptable level (> 0.340; see Weisburd and Britt, 2014), suggesting low collinearity amongst the predictor variables. At the first step of the analysis, age, sex and insomnia symptoms were significantly associated with increased body image disturbance. The addition of body image cognitive distortion, coping processes (i.e. appearance fixing, rationale acceptance, coping avoidance) and sleep-related monitoring at the second step of the analysis significantly increased predicted variance of body image disturbance by 38.9% (F change = 115.13, *p* < 0.001) and turned the effects of demographic variables non-significant. The significant correlates of insomnia symptoms at the second step included insomnia symptoms, body image cognitive distortions, coping avoidance, rationale acceptance, appearance fixing and awakening monitoring. The findings from the regression analysis are summarized in Table 2.

**Table 2:** Multivariate associations between insomnia symptoms, sleep-associated monitoring and body image disturbance variables

Predictors	Adjusted *R*^2^	*β*	B	Sig	95% CIs for B
*Step 1*	0.206				
Age		− 0.01	− 0.13	0.001***	− 0.015, − 0.005
Sex		0.40	0.13	0.001***	0.214, 0.599
Insomnia symptoms		− 0.05	− 0.42	0.001***	− 0.061, − 0.045
*Step 2*	0.593				
Age		0.00	0.01	0.517	− 0.003, 0.005
Sex		− 0.07	− 0.02	0.320	− 0.218, 0.071
Insomnia symptoms		− 0.01	− 0.07	0.024*	− 0.018, − 0.001
Body image cognitive distortion		0.39	0.44	0.001***	0.324, 0.465
Coping appearance fixing		0.24	0.17	0.000***	0.145, 0.336
Coping avoidance		0.24	0.16	0.001***	0.144, 0.342
Coping rationale acceptance		− 0.13	− 0.08	0.001***	− 0.219, − 0.056
Monitoring: awakening		0.07	0. 08	0.027*	0.009, 0.148
Monitoring: daytime		0.00	− 0.00	0.989	− 0.066, 0.065

Bootstrapped with 1000 resamples

*Sig at ≤ 0.05, ** ≤ 0.01, *** ≤ 0.001

**Fig. 1:**
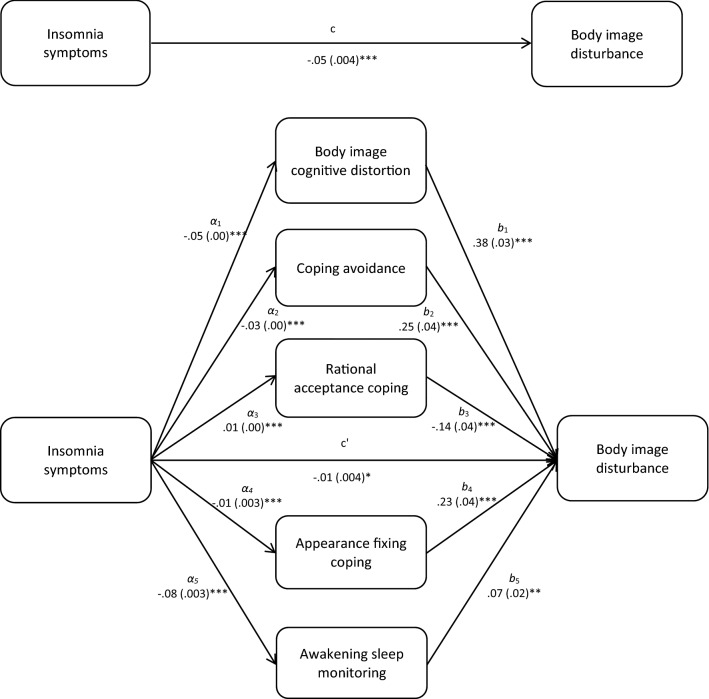
Indirect association of insomnia symptoms with body image disturbance, via body image-related cognitive distortions and coping, and sleep monitoring. The total (c) and the indirect effect (c’) of insomnia symptoms on body image disturbance. Unstandardized path coefficients are presented, with standard errors in brackets; *p < 0.05; **p < 0.005; ***p < 0.001

Multiple mediation modelling was used using Preacher et al. (2007) and Hayes (2013) recommendations. Specifically, bootstrapping with 1000 resamples and 95% and bias-corrected and accelerated confidence intervals were used. One multiple mediation model was computed to examine the indirect association of insomnia symptoms with body image disturbance, via body image cognitive distortions, coping avoidance, rationale acceptance coping, appearance fixing coping and awakening sleep monitoring (Fig. 1). Daytime sleep monitoring was not included in the mediation model because it had a non-significant association with the criterion variable (i.e. body image disturbance) in the multivariate model. The results showed that the association between insomnia symptoms and body image disturbance was mediated by body image cognitive distortions (*z* = − 7.70, *p* < 0.001), coping avoidance (*z* = − 4.95, *p* < 0.001), rationale acceptance coping (*z* = − 2.93, *p* = 0.003), appearance fixing coping (*z* = − 3.76, *p* < 0.001) and awakening sleep monitoring (*z* = − 2.69, *p* = 0.007).

## Discussion

The present study examined whether sleep-associated monitoring and negative coping strategies added incremental variance to, and also explained the multivariate association between different measures of body image disturbance (BID) and insomnia symptoms in adults. To the best of the authors’ knowledge, this is the first study to examine the relationship between sleep disturbance and body image concerns related to one’s appearance, with consideration of cognitive mechanisms, in members of the general population. Previous research with a community sample of young (aged 18–23 years) women in Spain, Seigel and colleagues (2004) demonstrated a positive relationship between self-reported sleep disturbances and attitudinal body image concerns including a fear of gaining weight, feeling overweight and dissatisfaction with the body. Additionally, in pregnant women, poor sleep quality has previously been associated with negative body attitudes (i.e. perceived weight, attractiveness, strength and fitness, salience of weight and shape). Our results expand on this existing research by providing novel evidence that specific aspects of body image disturbance and coping are associated with symptoms of insomnia. In relation to the first aim, symptoms of insomnia and sleep-associated monitoring behaviour were independently related to increased reports of body image disturbance, cognitive distortions of body image, appearance fixing (i.e. altering appearance by covering, camouflaging or correcting the perceived defect), avoidance (i.e. attempt to escape or avert stressful body image situations) and reduced levels of positive rationale acceptance (i.e. acceptance of the challenging event and positive self-care or rationale self-talk about one’s appearance). These results expand on previous examination of cutaneous body image, by highlighting body image disturbance (i.e. perception of physical defects) to be associated with symptoms of insomnia (Akram 2017; Gupta et al. 2015; Oyetakin-White et al. 2015).

In relation to our second aim, sleep-associated monitoring on awakening, cognitive distortion of body image and negative coping strategies related to body image (i.e. appearance fixing, avoidance, rationale acceptance), mediated the relationship between reports of body image disturbance and insomnia symptoms. From a cognitive perspective, the underlying mechanisms of insomnia and body image disturbance are similar, yet oriented towards each respective problem (i.e. sleep and/or body image). Indeed, the experience of insomnia and body image disturbances are partly developed and maintained by disordered processing of sleep and appearance-related information or events (Cash 2002; Harvey 2002). In this context, distortions (or biases) involve cognitive factors which alter the self-appraisal of sleep (i.e. sleep-associated monitoring of bodily sensations on waking for physical signs of a poor night’s sleep, fatigue and tiredness) and the physical self (i.e. negative interpretation of physical cues). Following, particular coping strategies may be deployed to manage distressing thoughts, negative feelings and behavioural responses related to BID and insomnia (Cash 2002; Harvey 2002; Snyder and Dinoff 1999). In the context of body image, inadequate strategies such as avoidance and reduced positive rationale acceptance may serve to paradoxically increase and further internalize body image disturbance. In this case, appearance fixing possibly maintains selective attention for physical cues and appearance-related stimuli (Cash and Labarge 1996; Cash et al. 2004), whereas avoidance of stressful body image situations likely facilitates the experience of social isolation, loneliness and depression (Angrish and Dhilon 2020; Cattarin and Thompson 1994; Laursen and Hartl 2013; Stice et al. 2000). Eventually, negative self-appraisal may transition into an actual body image disturbance characterized by body image dissatisfaction, distress (or dysphoria) and dysfunction (or impairment: Cash 2011). In a similar manner, inadequate strategies deployed to resolve poor sleep such as behavioural sleep efforts (e.g. daytime napping, going to sleep earlier than usual), avoidance of social and work commitments (e.g. “I look and feel too tired, I will stay home and rest”) and safety behaviours (e.g. appearance fixing due to perceived tiredness) serve to perpetuate the experience of insomnia (Harvey 2002). Therefore, the co-occurrence of insomnia symptoms and body image disturbances may increase the probability that the aforementioned mechanisms interact to perpetuate each problem.

Whilst directionality cannot be inferred from the current cross-sectional outcomes, from a physiological perspective, it is theoretically plausible that persistently poor sleep objectively compromises one’s physical appearance. In turn, this may contribute to the experience of body image disturbances in some individuals. Over time, the experience of poor and deprived sleep promotes HPA axis activation and modulation of the immune system, compromising skin barrier function, prompting inflammatory responses and the onset and/or exacerbation of skin disorders including psoriasis, acne and atopic dermatitis (Theoharides et al. 1998; Chiu et al. 2003; Hänel et al. 2013). Considering this, many dermatopathologies are related to disturbed sleep (e.g. psoriasis, acne, Duffin et al. 2009; Gupta et al. 2016; Schrom et al. 2019). For example, in a sample of Japanese students, increased reports of stress-related sleep disturbances were believed to accentuate their acne (Kubota et al. 2010). Similarly, increased reports of insomnia symptoms are reported amongst patients with psoriasis (Shutty et al. 2013). This latter observation, however, may be explained by bouts of itching through the nocturnal sleep period which impairs sleep through the precipitation of increased arousals that fragment objective sleep continuity (Aoki et al. 1991; Savin et al. 1990). Whilst the experience of insomnia may lead to dermatological changes, the emergence of any major physical alterations to the rest of the body remains unlikely. An alternative possibility is that the experience of body image disturbances leads to poor sleep and symptoms of insomnia. Here, daytime selective attention towards the physical body may prompt increased worry and rumination during the pre-sleep period, whereas socially avoidant, and potentially sedentary, behaviours may reduce the homeostatic drive for sleep. Both of them may contribute to a delay in sleep onset latency. To date, research in this area has focused solely on the relationship between body mass index (BMI), eating behaviour and disturbed sleep, rather than the self-perception of body image. Here, self-reported sleep disturbances appear to be consistently more prominent amongst over and underweight individuals (Siversten et al. 2014; Soares et al. 2011; Vargas et al. 2014). Moreover, examination of sleep duration in this context demonstrates that high BMI individuals experience the shortest sleep, whereas low BMI individuals report the longest sleep duration (Grandner et al. 2015). Nevertheless, further longitudinal research should aim to tease apart the directionality to the insomnia and body image disturbance relationship.

A number of limitations of the current study should be noted. The cross-sectional design and target sample and sample size may leave the current outcomes vulnerable to inflation bias between variables and limit the ability to draw conclusions about causal relationships. As the present sample was comprised mostly of females, further research using a more balanced sample should clarify the role of sex. Longitudinal examination of the current research questions should clarify whether body image disturbance predicts insomnia symptoms, or whether the reverse is true. Finally, further research may examine the role of commonly presenting comorbid psychiatric symptoms (e.g. anxiety, depression).

Taken together, the current outcomes highlight the relationship between measures of body image disturbance (BID) and insomnia symptoms. More crucially, sleep-associated monitoring and specific body image coping mechanisms mediated the relationships between body image disturbance measures and insomnia symptoms. Our results highlight the role of cognitive and behavioural mechanisms pertaining to sleep (i.e. selective attention for physical signs of poor sleep) and body image (i.e. behavioural avoidance, appearance fixing and selective attention) which may be targeted as part of cognitive behavioural treatments. Interventions aiming to reduce the risk of body image disturbance may consider addressing the role of (poor) sleep and sleep-associated monitoring as an adjunct target. Here, cognitive components of the cognitive behavioural therapy for insomnia (CBTi) curriculum may serve to reduce behavioural sleep efforts, safety behaviours, selective attention, catastrophizing and worry about sleep and the consequences of poor sleep whilst also correcting dysfunctional sleep-related cognition in this population.

## Data Availability

Data will be made available on reasonable request.
